# Unique mutation spectrum of progressive pseudorheumatoid dysplasia in the Chinese population: a retrospective genotype–phenotype analysis of 105 patients

**DOI:** 10.1007/s12519-022-00674-7

**Published:** 2023-01-09

**Authors:** Wei Wang, Si-Hao Gao, Min Wei, Lin-Qing Zhong, Wei Liu, Shan Jian, Juan Xiao, Cai-Hui Zhang, Jian-Guo Zhang, Xiao-Feng Zeng, Wei-Bo Xia, Zheng-Qing Qiu, Hong-Mei Song

**Affiliations:** 1 Department of Pediatrics, Peking Union Medical College Hospital, Chinese Academy of Medical Sciences and Peking Union Medical College, No. 1 Shuaifuyuan, Dongcheng, Beijing 100730, China; 2 Department of Radiology, Peking Union Medical College Hospital, Chinese Academy of Medical Sciences and Peking Union Medical College, Beijing 100730, China; 3 Department of Orthopedic Surgery, Peking Union Medical College Hospital, Chinese Academy of Medical Sciences and Peking Union Medical College, Beijing 100730, China; 4 Department of Rheumatology and Clinical Immunology, Chinese Academy of Medical Sciences and Peking Union Medical College, Beijing 100730, China; 5 National Clinical Research Center for Dermatologic and Immunologic Diseases (NCRC-DID), Ministry of Science and Technology, Beijing 100730, China; 6 State Key Laboratory of Complex Severe and Rare Diseases, Peking Union Medical College Hospital (PUMCH), Beijing 100730, China; 7 Key Laboratory of Rheumatology and Clinical Immunology, Ministry of Education, Beijing 100730, China; 8 Department of Endocrinology, National Health Commission Key Laboratory of Endocrinology, Peking Union Medical College Hospital, Chinese Academy of Medical Sciences and Peking Union Medical College, Beijing 100730, China

**Keywords:** *CCN6*, Genetics, Mutations, Progressive pseudorheumatoid dysplasia, *WISP3*

## Abstract

**Background:**

Progressive pseudorheumatoid dysplasia (PPRD) is a rare genetic disease with autosomal recessive inheritance. There was a lack of genotype–phenotype correlation data from the Chinese population. This study aimed to identify the genotype and phenotype characteristics of Chinese PPRD patients and to conduct a genotype–phenotype analysis of Chinese PPRD patients.

**Methods:**

Genetic analysis was performed for suspected PPRD patients from Peking Union Medical College Hospital. Medical records were collected from the electronic medical record system and patient-held portable health records. Published Chinese PPRD cases were gathered from both international and Chinese local databases. We collected demographic information, genetic variants, clinical manifestations, and imaging characteristics for further analysis.

**Results:**

We included 105 Chinese PPRD patients in the current study. Thirty-three variants, including nine novels and five hotspot variants, were identified, with 26/33 (79%) variants exclusively seen in the Chinese population. Chinese PPRD patients share a phenotype similar to that in international reports. Joint involvement may progress with age (*R*^2^ = 0.2541). Long bone shortening and severe deformities occur in three patients with biallelic null variants, of which at least one variant is located in exon 2. Among hotspot variants, c.624dupA (p.C209Mfs*21) were associated with later onset and more involved joints. Elbow joints were more likely to be affected in patients carrying c.624dupA (p.C209Mfs*21) and c.866dupA (p.S209Efs*13). Shoulder joints are more likely to be involved in patients with biallelic null variants (*P* = 0.027).

**Conclusions:**

Chinese PPRD patients share a unique mutation spectrum. Among the five hotspot variants, c.624dupA is associated with later onset of disease, more extensive joint involvement, and a tendency to affect elbow joints. Biallelic null variants with at least one variant in exon 2 could be a likely cause of long bone shortening and severe deformities.

**Supplementary Information:**

The online version contains supplementary material available at 10.1007/s12519-022-00674-7.

## Introduction

Progressive pseudorheumatoid dysplasia (PPRD), initially described in 1982 [1], is a rare autosomal recessive disorder characterized by spondyloepiphyseal dysplasia. The causal gene *CCN6* (cellular communication network factor 6 gene), previously known as *WISP3* (WNT1-inducible signaling pathway protein-3 gene), was first cloned and sequenced in 1998 [2]. The first seven Chinese cases were reported in 1986 without genetic confirmation [3]. Due to the low incidence of PPRD, currently published case reports or series often consist of only a few patients [4–6]. Neither large cohort analyses nor epidemiological surveys of PPRD have been conducted in China. To summarize the genotypic and phenotypic features of PPRD in the Chinese population, we report a single-center cohort of 61 patients genetically diagnosed with PPRD from Peking Union Medical College Hospital (PUMCH) and conduct a retrospective analysis of 44 genetically confirmed Chinese PPRD cases by searching the literature before October 2021.

## Methods

### Patients

This retrospective study included all patients with suspected PPRD seen at the genetic clinics of the Pediatric Department, PUMCH between January 2007 and October 2021. The inclusion criteria were as follows: (1) progressive polyarticular deformities with a limited range of motion (involving bilateral interphalangeal joints, elbows, knees, or hips) or progressive bone malformations, with varying degrees of severity and sequences of progression; (2) with or without spinal deformities such as scoliosis and kyphosis; and (3) no fever and rashes. The exclusion criteria were as follows: (1) persistent elevation of inflammatory markers, such as erythrocyte sedimentation rate (ESR) and C-reactive protein (CRP); and (2) other monogenic diseases presenting with spondyloepiphyseal dysplasia.

Inpatient and outpatient medical records were collected from patient-held portable health records and the electronic medical record system of PUMCH. Potential cases with *WISP3* or *CCN6* variants were collected, followed by genetic confirmation. This study was approved by the Ethics Committee of PUMCH (S-609).

### Literature-derived patients

We searched PubMed, Wanfang Data, China Science and Technology Journal Database (CQVIP), China Knowledge Resource Integrated Database (CNKI), and Chinese Medical Journal Network by using combinations of the following keywords: “progressive pseudorheumatoid dysplasia”, “spondyloepiphyseal dysplasia”, “*WISP3*”, “*CCN6*”, “Chinese”, and “China”. We put no restrictions on the publication dates. Cases with no genetic confirmation were excluded. We also removed duplicate cases in different studies and the cases overlapping our PUMCH cohort. Cases with detailed clinical data were selected for further analysis of their demographic information, genetic mutations, clinical manifestations, and radiological characteristics.

### Genetic analysis

Sanger sequencing was performed at the PUMCH laboratory for all 42 PUMCH patients with suspected PPRD between 2007 and 2013. Targeted next-generation sequencing (NGS) panel or Sanger sequencing was applied by third-party companies for the remaining PUMCH patients from 2014 to date.

#### Sanger sequencing

We obtained peripheral blood from PUMCH patients and/or their parents, followed by DNA extraction. Polymerase chain reaction (PCR) primers (Supplementary Table 1) were designed with Primer3.0 online software. We performed PCR amplification and product purification. Sanger sequencing was then applied to sequence the five exons of *WISP3* as well as exon‒intron boundaries.

#### Targeted next-generation sequencing

Peripheral blood was obtained from PUMCH patients and/or their parents for targeted NGS by third-party companies. The general steps include DNA extraction, DNA library preparation, enrichment and sequencing of targeted genes, and bioinformatic analysis. The sequencing results were validated with Sanger sequencing using primers designed by third-party companies.

### Statistical analysis

For descriptive statistics, median and range were used for numerical data that may not follow a normal distribution. The arithmetic mean and standard deviation were calculated for normally distributed numerical data. Categorical data were described with frequencies and proportions. Statistical inferences were made by SPSS 17.0 software. For hypothesis testing of normally distributed numerical data among multiple groups, we performed one-way ANOVA under the equal variance hypothesis. If the null hypothesis was rejected, the least significant difference *t* test approach was then applied to identify the significantly different pairs. If variances were heterogeneous across groups, Tamhane’s multiple comparison procedure was followed for intergroup analysis. For categorical data, Chi-square tests and Fisher’s exact test were applied. To compare multiple groups, we weighed the data by frequency and applied Fisher’s exact tests pair by pair. The threshold of statistical significance was set as a *P* value < 0.05.

## Results

### Genetic testing results

#### Genetic testing of PPRD patients from PUMCH

**Fig. 1:**
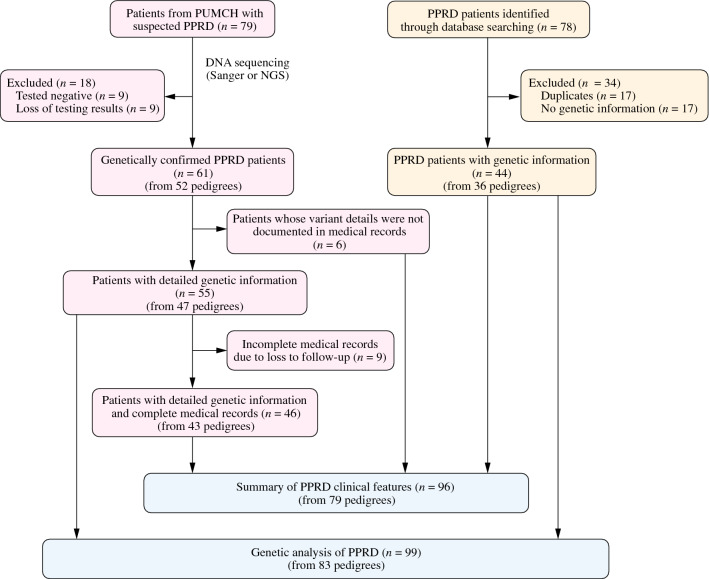
Flowchart of patient collection. *PUMCH* Peking Union Medical College Hospital, *PPRD* progressive pseudorheumatoid dysplasia, *NGS* next-generation sequencing

Genetic analysis of our PUMCH cohort was conducted on 55 Chinese patients from 47 pedigrees (Fig. 1). Briefly, we enrolled and conducted genetic tests on 79 patients with suspected PPRD from the Department of Pediatrics, PUMCH. We successfully diagnosed 61 patients from 52 pedigrees in our center, of which six patients from five pedigrees were excluded from genetic analysis due to a lack of variation details from their medical records.

Biallelic compound heterozygous or homozygous *CCN6* variants were identified in 52 patients, including one patient who carried three *CCN6* variants. Three patients were heterozygous for *CCN6*. In sum, twenty *CCN6* variants were identified from the 55 patients, including nine novel variants (NM_198239.2): c.135dupA (p.Q46Tfs*31); c.166C>T (p.Q56*); c.238A>G (p.K80E); c.250delG (p.E84Kfs*21); c.419dupT (p.L140Ffs*30); c.556C>T (p.Q186*); c.625T>A (p.C209S); c.802T>C (p.C268R); c.1012C>T (p.Q338*).

#### *CCN6* mutations in Chinese PPRD patients

After a literature search, forty-four literature-derived patients from 36 pedigrees were included in this study (Fig. 1). Twenty-four variants were collected from the literature, including 11 variants also observed in our PUMCH cohort. Biallelic *CCN6* variants were found in 39 patients. The remaining five patients carried only one monoallelic variant.

**Fig. 2:**
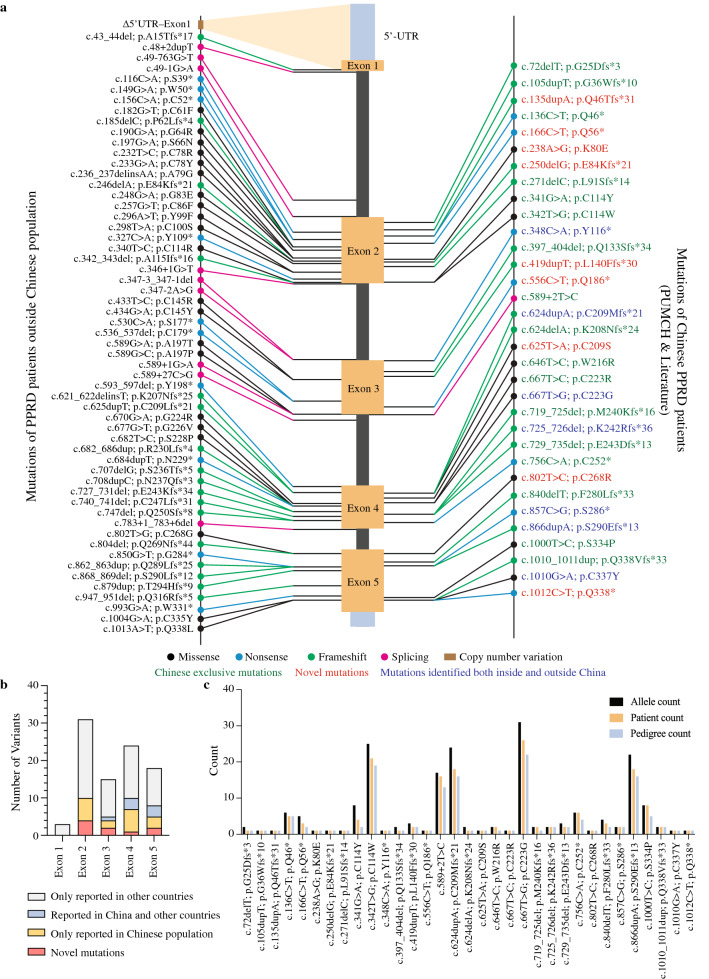
Frequency and distribution of *CCN6* mutations. **a** Comparison of Chinese *CCN6* variants (right panel) with non-Chinese variants from HGMD (left panel); **b** exon distribution of Chinese and non-Chinese variations; **c** hotspot variants in Chinese cohort as revealed by allele, patient, and pedigree counts. *CCN6* cellular communication network factor 6 gene, *PUMCH* Peking Union Medical College Hospital, *PPRD* progressive pseudorheumatoid dysplasia, *HGMD* Human Gene Mutation Database

By combining PUMCH-identified variants with those of 44 genetically confirmed PPRD patients from the literature, we gathered 33 variants in 99 PPRD patients from 83 Chinese pedigrees (Fig. 2). All variants were located in exons 2–5 of *CCN6* (NM_198239.2). Among the 33 variants, there were 15 frameshift, 10 missense, and seven nonsense variants, as well as one splicing variant (Table 1). The hotspot variants were c.667T>G (p.C223G), c.624dupA (p.C209Mfs*21), c.342T>G (p.C114W), c.866dupA (p.S290Efs*13), and c.589+2T>C. Nine of the 33 variants were novel variants. Among the previously reported variants, twenty variants have been recorded in the Human Gene Mutation Database (HGMD), with only seven reported in patients outside China. In sum, there are 26 variants exclusively seen in Chinese patients.

**Table 1:** Genetic analysis of progressive pseudorheumatoid dysplasia

cDNA alteration	Amino acid alteration	ACMG	Recorded in HGMD	Chinese exclusive	Exon	Allele count^a^	Patient count^a^	Pedigree count^a^	Source
c.72delT	p.G25Dfs*3	P	Yes	Yes	2	2	1	1	[7]
c.105dupT	p.G36Wfs*10	P	Yes	Yes	2	1	1	1	[8]
c.135dupA^b^	p.Q46Tfs*31	P	No	Yes	2	1	1	1	Current study
c.136C>T	p.Q46*	P	Yes	Yes	2	6	5	5	Current study, [4, 9–11]
c.166C>T^b^	p.Q56*	P	No	Yes	2	3	3	2	Current study
c.238A>G^b^	p.K80E	LP	No	Yes	2	1	1	1	Current study
c.250delG^b^	p.E84Kfs*21	P	No	Yes	2	1	1	1	Current study
c.271delC	p.L91Sfs*14	P	No	Yes	2	1	1	1	[4]
c.341G>A	p.C114Y	P	Yes	Yes	2	4	4	2	[10, 12]
c.342T>G	p.C114W	P	Yes	Yes	2	23	21	19	Current study, [8, 9, 11, 13–15]
c.348C>A	p.Y116*	P	Yes	No	3	1	1	1	[13]
c.397_404del	p.Q133Sfs*34	P	No	Yes	3	2	1	1	[16]
c.419dupT^b^	p.L140Ffs*30	P	No	Yes	3	3	2	2	Current study
c.556C>T^b^	p.Q186*	P	No	Yes	3	1	1	1	Current study
c.589+2T>C	–	LP	Yes	Yes	3	14	16	13	Current study, [4, 13, 16]
c.624dupA	p.C209Mfs*21	P	Yes	No	4	21	18	16	Current study, [9, 13, 14, 17–19]
c.624delA	p.K208Nfs*24	P	Yes	Yes	4	2	1	1	[8]
c.625T>A^b^	p.C209S	LP	No	Yes	4	1	1	1	Current study
c.646T>C	p.W216R	LP	No	Yes	4	2	2	1	[16]
c.667T>C	p.C223R	LP	No	Yes	4	1	1	1	[13]
c.667T>G	p.C223G	P	Yes	No	4	26	26	22	Current study, [4, 9, 11, 14, 16, 20]
c.719_725del	p.M240Kfs*16	P	Yes	Yes	4	1	2	1	[15]
c.725_726del	p.K242Rfs*36	P	Yes	No	4	3	2	2	Current study, [21]
c.729_735del	p.E243Dfs*13	P	Yes	Yes	4	2	2	2	[17, 22]
c.756C>A	p.C252*	P	Yes	Yes	4	4	6	4	Current study, [18, 20, 23]
c.802T>C^b^	p.C268R	LP	No	Yes	5	1	1	1	Current study
c.840delT	p.F280Lfs*33	P	Yes	Yes	5	3	3	2	[24, 25]
c.857C>G	p.S286*	P	Yes	No	5	2	2	2	Current study, [11]
c.866dupA	p.S290Efs*13	P	Yes	No	5	19	18	16	Current study, [11, 17, 23]
c.1000T>C	p.S334P	P	Yes	Yes	5	5	8	5	Current study, [16, 24]
c.1010_1011dup	p.Q338Vfs*33	P	Yes	Yes	5	2	2	2	Current study, [19]
c.1010G>A	p.C337Y	P	Yes	No	5	1	1	1	[19]
c.1012C>T^b^	p.Q338*	P	No	Yes	5	1	1	1	Current study

*P* pathogenic, *LP* likely pathogenic, *ACMG* American College of Medical Genetics, *HGMD* Human Gene Mutation Database. ^a^Ninety-nine patients with 161 mutated alleles from 83 pedigrees were included in the genetic analysis (8 patients had only one heterozygous variant; one patients had three variants); ^b^novel variants

### Clinical manifestations

After excluding poorly documented cases, duplicate ones, and those without genetic confirmation, ninety-six patients from 79 pedigrees were included in the clinical analysis, including 52 cases diagnosed by PUMCH and 44 cases retrieved from the literature (Fig. 1).

#### PPRD patients from PUMCH

The clinical analysis was conducted on 52 of the 61 genetically diagnosed patients, as nine patient-held portable medical records could not be retrieved. The 52 PUMCH-diagnosed PPRD patients, including 32 males and 20 females, came from 48 pedigrees. The median age of initial consultation at PUMCH was 12 years (range: 3–34), with 79% (41/52) under 18. The median age of onset was 6 years (range: 0.5–29). The dates of genetic diagnosis were documented in 35 patients. The median gap between disease onset and the genetic diagnosis was 6 years (range: 1–29). Thirteen patients were documented to have PPRD siblings. Consanguineous marriage was documented in four patients from three pedigrees. Ethnic information was recorded in 27 patients, all of whom were Han Chinese.

All patients were intellectually normal. Most patients were characterized by short stature, progressive deformities in multiple joints with limited range of motion and tenderness. The laboratory investigations returned all normal for complete blood count, biochemistry profile, ESR, CRP, and autoantibodies.

#### Chinese PPRD patients combined

*Demographic information* Combining PUMCH and literature-identified PPRD cases, the median age of onset was 6 years (range: 0.5–29). Of the 93 patients with documented initial symptoms, 72% (67/93) experienced their initial symptoms before seven. Only two patients had disease onset in adulthood, at 24 and 29 years old. The median age of initial consultation (at PUMCH or as reported from the literature) was 14 years (range: 2–59). Height was documented in 43 patients below 18 years old, with 70% (30/43) below the 3rd percentile. Weight was documented in 33 patients below 18 years old, with 33% (11/33) below the 3rd percentile. Seventy-one percent (12/17) of the adult patients with documented height were below the 3rd percentile (references were taken from the same gender 18-year-old healthy Chinese population). Forty-two percent (5/12) of adults were below the 3rd percentile in weight (reference as above). The proportions of short stature (height < 3rd percentile) were not significantly different in adult and pediatric patients (*P* = 1.000).

*General signs* Collectively, the 96 Chinese PPRD patients presented with short stature, short neck, thoracic deformities, short trunk, polyarticular joint enlargement and flexion deformities, spinal curvature, pronounced pelvic tilt in the standing position, and progressive gait changes ranging from normal gait to limping gait to waddling gait. In severe cases, patients were wheelchair-dependent and unable to walk at all. Muscular atrophy was observed in wheelchair-dependent patients. Seven patients were documented to use wheelchairs to enter the clinic, with ages ranging from 11 to 17 years.

**Fig. 3:**
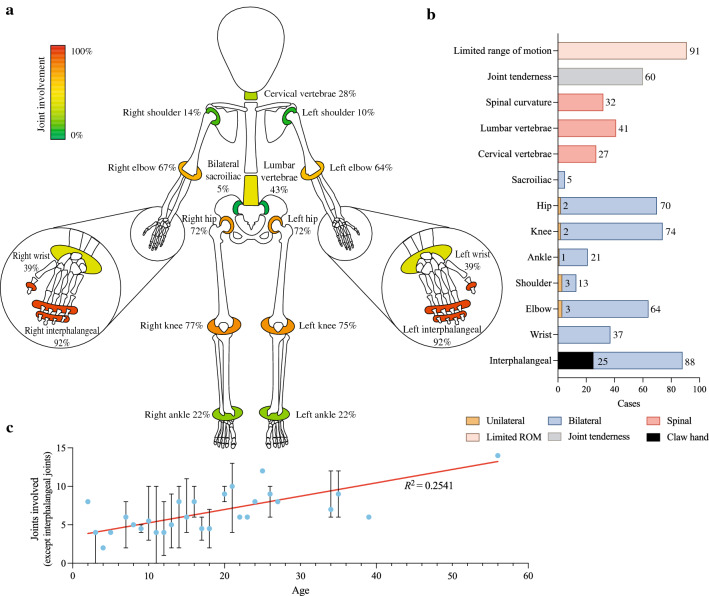
Joint involvement of progressive pseudorheumatoid dysplasia (PPRD). **a, b** Frequencies of the joints involved in Chinese PPRD cohort; **c** positive correlation between affected joints and age. *ROM* range of motion

*Joint involvement* PPRD showed a distinct pattern of joint involvement (Fig. 3a). Bilateral interphalangeal joints were involved in 92% (88/96) of cases, including 25 cases of flexion deformities or claw hands. Knees were affected in 77% (74/96) of cases, including 72 cases involving bilateral knees and two cases unilaterally affecting the right knee. Hip joints were involved in 73% (70/96) of cases, including 68 bilaterally involved, one unilaterally right-sided, and one unilaterally left-sided. Elbows were affected in 67% (64/96) of patients, including 61 bilaterally involved patients and three unilaterally right-sided patients. Bilateral wrists were involved in 39% (37/96) of patients. The shoulder joint was involved in 14% (13/96) of patients (ten bilaterally and three unilaterally right-sided). Sacroiliac joints were involved in 5% (5/96) of patients. Interphalangeal joint involvement of the foot was extremely rare, as there were only four documented cases (three bilaterally and one unilaterally right-sided). For the axial skeleton, lumbar vertebrae were involved in 43% (41/96) of patients. The cervical spine was affected in 28% (27/96) of patients. Thirty-two (33%) patients had spinal curvature. Limited range of motion was documented in 95% (91/96) of patients, and 62% (60/96) of patients experienced joint tenderness (Fig. 3b).

A 3-year-old boy with no joint involvement had bilateral symmetrical curvature of the tibia and fibula. An 11-year-old boy had only enlargement of bilateral interphalangeal joints of the hand and mild pectus carinatum. The number of involved joints (excluding interphalangeal joints) seemed positively correlated with age (Fig. 3c).

**Fig. 4:**
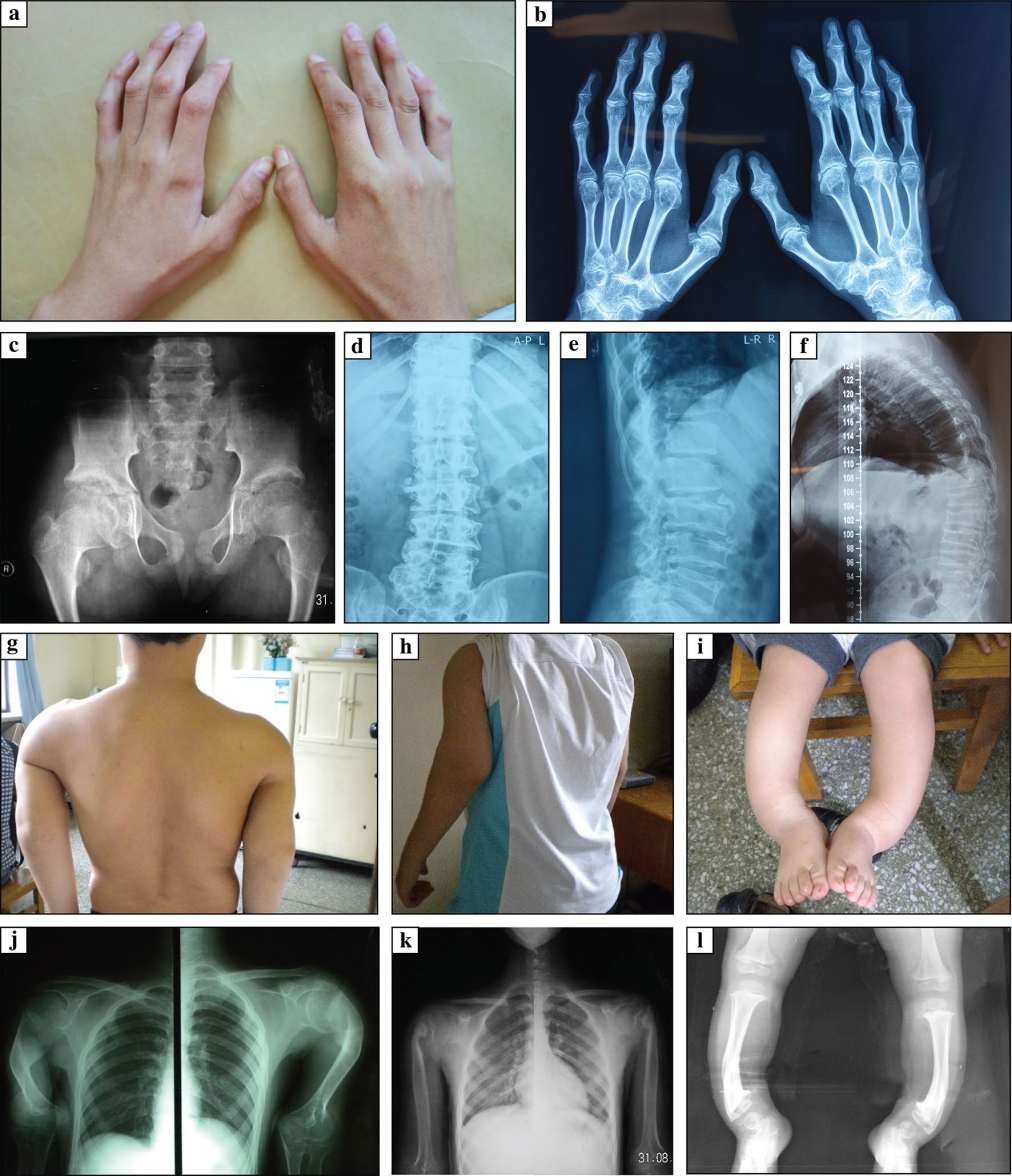
Clinical and Imaging findings of progressive pseudorheumatoid dysplasia (PPRD). Typical imaging findings of PPRD (**a–f**) and imaging findings of PPRD cases with atypical severe long bone involvement (**g**–**l**). **a**, **b** Flexion deformity and claw shape of bilateral hands. X-ray showed osteoporosis of bilateral hands, epiphyseal enlargement of the metacarpophalangeal, proximal and distal interphalangeal joints, narrowing of the metacarpophalangeal joint spaces, irregularity of articular surfaces, and joint deformities (case 1); **c** widening of the pubic symphysis and bilateral sacroiliac joint spaces. Osteoporosis and degeneration of the bilteral hip joints, narrowed hip joints spaces, and ill-defined articular surfaces can be observed. Cystic changes were under the articular surfaces of bilateral femoral heads. The femoral necks were also shortened (case 2); **d**–**f** scoliosis or kyphosis of the spine (**d** and **e** from case 1; **f** from case 3). The vertebral bodies were flattened. There was a lack of regularity on the superior and inferior surfaces; **g**, **j** shortening and flexion deformities of the bilateral humeri with metaphyseal or epiphyseal enlargement of the upper and lower ends. The elbow joints were enlarged with narrowed joint spaces. There might be a rotational deformity of the humerus on the right shoulder (case 4); **h**, **k** enlargement of the humeral head. The humeral neck was narrowed. There might be a flexion deformity (case 1); **i**, **l** varus deformities of the bilateral lower extremities. The tibia and fibula on the bilateral sides were symmetrically curved. There were metaphyseal enlargements of the knees and ankles, and a widening of the joint spaces (case 5, the younger brother of case 1)

*Imaging findings* As revealed by X-ray or computed tomography scans, PPRD patients typically showed osteoporosis, polyarticular joint enlargement and degeneration, joint space narrowing, irregular articular surfaces, and dystrophy of the epiphyses or metaphyses (Fig. 4). Imaging findings of bilateral hands include thickening and enlargements of metaphyses, as well as narrowing, enlargement, and flexion deformities of interphalangeal joints. Spinal findings include narrowed anterior borders and abnormally flattened vertebral bodies, in addition to scoliosis, kyphosis, or lordosis. Severe long bone deformities occurred in three patients from two pedigrees. Case 4 is an adult male with bilateral humeral shortening and flexion deformities. He carries biallelic compound heterozygous variants at c.135dupA (p.Q46Tfs*31) and c.866dupA (p.S290Efs*3). Another two cases are siblings with homozygous variants at c.166C>T (p.Q56*). The older sister is an adult female with humeral head enlargement, ahumeral neck narrowing, humeral bone shortening, and localized flexion deformities. The 3-year-old brother had an early onset of the disease 6 months after birth. He developed progressive varus deformities of the bilateral lower extremities, with symmetrical curvature of the tibia and fibula.

#### Association analysis between genotypes and clinical phenotypes

*The association between mutational hotspots and phenotypes* For genotype–phenotype analysis, we compared the age of onset, age at an initial consultation, gender ratio, number and patterns of joint involvement (excluding interphalangeal joints), percentage of spinal curvature, and joint symptoms (including a limited range of motion and arthralgia) in PPRD patients carrying the five hotspot variants (Table 2). Patients with c.624dupA (p.C209Mfs*21) variant had more involved joints than patients with the c.342T>G (p.C114W) and c.667T>G (p.C223G) variants (*P* = 0.013 and 0.016, respectively). Furthermore, elbow joints are more likely to be involved in patients carrying c.624dupA (p.C209Mfs*21) and c.866dupA (p.S290Efs*13) variants (*P* = 0.028).

**Table 2:** The associations between *CCN6* hotspot variants and clinical phenotype

Variables	Hotspot variants					*P*
	1	2	3	4	5	
	c.342T>G (p.C114W)	c.589+2T>C	c.624dupA (p.C209Mfs*21)	c.667T>G (p.C223G)	c.866dupA (p.S209Efs*13)	
Total cases	15	12	16	24	17	
Sex ratio (M/F)	7/8	7/5	7/9	14/10	12/5	0.549
Age (y), mean ± SD						
Onset	5.00 ± 2.42	5.98 ± 3.12	9.25 ± 4.58	6.25 ± 3.57	6.34 ± 4.27	0.034 (*P*_1–3_)
Initial consultation	15.60 ± 7.23	12.92 ± 2.39	20.56 ± 6.86	14.70 ± 5.36	18.35 ± 11.41	0.005 (*P*_2-3_)
No. of joints involved (except IPs)	5.29 ± 3.15	6.67 ± 3.14	8.14 ± 2.63	5.65 ± 3.10	7.06 ± 2.794	0.013 (*P*_1–3_) 0.016 (*P*_3–4_)
Joints involvement (affected/unaffected)						
Bilateral PIPs	14/1	12/0	13/3	22/2	16/1	0.586 (*P*_f_)
Hip	8/7	10/2	13/3	20/4	12/5	0.276 (*P*_f_)
Sacroiliac	0	1/11	1/15	1/23	2/15	0.920 (*P*_f_)
Shoulder	0	2/10	4/12	3/21	4/13	0.707 (*P*_f_)
Knee	13/2	8/4	13/3	16/8	15/2	0.384 (*P*_f_)
Ankle	4/11	4/8	5/11	2/22	5/12	0.258 (*P*_f_)
Elbow	8/7	8/4	13/3	11/13	15/2	0.028 (*P*_f_)
Wrist	4/11	6/6	7/9	8/16	5/12	0.684 (*P*_f_)
Cervical vertebrae	2/13	1/11	5/11	3/21	4/13	0.497 (*P*_f_)
Lumbar vertebrae	4/11	6/6	5/11	9/15	6/11	0.796 (*P*_f_)
Joint symptoms (affected/unaffected)						
Limited range of motion	14/1	12/0	15/1	23/1	17/0	0.867 (*P*_f_)
Arthralgia	8/7	8/4	14/2	15/9	9/8	0.217 (*P*_f_)
Spinal curvature	2/13	4/11	7/9	8/16	5/12	0.463 (*P*_f_)

*M* male, *F* female, *IPs* interphalangeals, *PIPs* proximal interphalangeals, *SD* standard deviation, *P*_*f*_ Fisher’s exact test

*Genotype–phenotype associations* Of the 90 patients with complete clinical and genetic variant data, thirty-three patients carried biallelic *CCN6* null variants. Eight biallelic null variant carriers had at least one variant in exon 2. We compared the clinical features of patients with different genotypes and found that biallelic null variants are more likely to involve shoulder joints (*P* = 0.027) and are associated with long bone shortening with severe deformities (*P* = 0.049). Further analysis revealed that long bone shortening with severe deformities was associated with a higher proportion of patients with biallelic null variants, including at least one variant located in exon 2 than other genotypes (*P* < 0.001) (Table 3).

**Table 3:** Genotype–phenotype associations in progressive pseudorheumatoid dysplasia patients

Variables	Genotypes					*P*
	1	0	2	3	4	
	Biallelic null	Genotypes excluding biallelic null	1 null + 1 missense	Biallelic missense	Monoallelic null or missense	
Total cases	33	57	27	22	8	
Sex ratio (M/F)	21/12	32/25	20/7	9/13	3/5	0.063 (*P*_f_) 0.514 (*P*_1–0_)
Age (y), mean ± SD						
Onset	6.50 ± 4.18	7.06 ± 5.10	7.39 ± 5.39	5.22 ± 2.72	11.57 ± 7.09	0.017 0.009 (*P*_1–4_) 0.034 (*P*_2–4_) 0.002 (*P*_3–4_) 0.988 (*P*_1–0_)
Initial consultation	17.03 ± 10.79	15.08 ± 7.60	15.92 ± 7.11	13.28 ± 7.70	19.00 ± 10.42	0.438 0.160 (*P*_1–0_)
No. of joints involved (except IPs)	7.12 ± 3.44	5.54 ± 2.95	6.07 ± 2.33	4.67 ± 3.00	6.00 ± 4.30	0.054 0.006 (*P*_1–3_) 0.319 (*P*_1–0_)
Joints involvement (involved/unaffected)						
Bilateral PIPs	30/3	53/4	25/2	21/1	7/1	0.800 (*P*_f_) 0.704 (*P*_1–0_)
Hip	25/8	45/12	23/4	15/7	7/1	0.512 (*P*_f_) 0.795 (*P*_1–0_)
Sacroiliac	3/30	2/55	0/27	1/21	1/7	0.262 (*P*_f_) 0.352 (*P*_1–0_)
Shoulder	8/25	4/53	2/25	1/21	1/7	0.144 (*P*_f_) 0.027 (*P*_1–0_)
Knee	28/5	44/13	22/5	16/6	6/2	0.667 (*P*_f_) 0.427 (*P*_1–0_)
Ankle	10/23	11/46	6/21	2/20	3/5	0.189 (*P*_f_) 0.302 (*P*_1–0_)
Elbow	26/7	35/22	19/8	10/12	6/2	0.077 (*P*_f_) 0.106 (*P*_1–0_)
Wrist	16/17	18/39	8/19	5/17	5/3	0.092 (*P*_f_) 0.121 (*P*_1–0_)
Cervical vertebrae	13/20	14/43	3/24	6/16	5/3	0.014 (*P*_f_) 0.158 (*P*_1–0_)
Lumbar vertebrae	14/19	25/32	13/14	9/13	3/5	0.937 (*P*_f_) 0.536 (*P*_1–0_)
Joint symptoms (affected/unaffected)						
Limited range of motion	33/0	53/4	25/2	21/1	7/1	0.592 (*P*_f_) 0.292 (*P*_1–0_)
Arthralgia	25/8	35/22	13/14	16/6	6/2	0.132 (*P*_f_) 0.246 (*P*_1–0_)
Spinal curvature	11/22	19/38	8/19	6/16	3/5	0.925 (*P*_f_) 1.000 (*P*_1–0_)
Long bone shortening with severe deformities	3/30	0/57				0.049 (*P*_1–0_)
Long bone shortening with severe deformities	3/5^a^	0/83				˂ 0.001 (*P*_1–0_)

*M* male, *F* female, *IPs* interphalangeals, *PIPs* proximal interphalangeals, *SD* standard deviation, *P*_*f*_ Fisher’s exact test. ^a^Biallelic null variants with at least one null variant in exon 2

## Discussion

Progressive pseudorheumatoid arthritis (PPRD) is a rare progressive disease with spondyloepiphyseal dysplasia and an estimated incidence of 1/1,000,000 in the UK. No incidence rate data have been reported in China. The typical age of onset is 3–8 years [26, 27]. In this study, we conducted a large retrospective analysis of Chinese PPRD patients and showed for the first time that Chinese PPRD patients share a unique *CCN6* mutation spectrum with five hotspots. Typical and atypical PPRD phenotypes were characterized, and potential genotype–phenotype associations were identified.

Chinese patients shared a unique *CCN6* mutation spectrum (Fig. 2). As of April 2021, 78 PPRD-associated variants have been recorded by HGMD, including 13 Chinese-exclusive variants. Collectively, we identified a total of 33 variants widely distributed on *CCN6* exons 2–5 among the Chinese patients. Only seven variants have been reported outside China. Seventy-nine percent (26/33) of variants were exclusively seen in the Chinese population, indicating a unique variant spectrum in the Chinese ethnic group. Nine novel *CCN6* variants were also discovered. Among the novel variants, six were ACMG (American College of Medical Genetics) pathogenic null variants, including three frameshift and three nonsense variants that may trigger premature termination of protein translation. The remaining three novel variants are likely pathogenic missense variants. Five hotspot variants were identified.

We identified typical and atypical presentations as well as top joints involved in PPRD (Figs. 3, 4). Clinical features include abnormal gaits, fatigue, polyarticular joint deformities, arthralgia, and limited range of motion. The most commonly involved joints were bilateral interphalangeal joints of the hand, especially the distal joints, which can be considered a hallmark of this disease. The interphalangeal joints were followed by knees, hips, and elbows. Notably, almost all the cases showed symmetrical joint involvement, with only a few cases unilaterally affected. The unilaterally involved joints were more commonly positioned on the right side, probably associated with right-handedness. We observed a positive correlation between increased joint involvement with age, which was compatible with the progressive nature of this disease. Chinese PPRD patients presented with those from other countries. About 70% of Chinese PPRD patients’ heights were below the 3rd percentile, showing no significant differences between adult and pediatric patients.

A later age of onset may be associated with c.725_726del and c.624dupA. In our cohort, only two patients had their disease onset in adulthood, all carrying c.725_726del. One patient [21] was 24 years old at the onset with a single maternal-derived monoallelic variant c.725_726del (p.K242Rfs*36). Another case was a 29-year-old male who carried biallelic compound heterozygous mutations of c.625T>A (p.C209S) and c.725_726del (p.K242Rfs*36). In addition, c.624dupA was found to be associated with later onset by comparison among hotspot variants.

Patients with c.624dupA and biallelic null variants are likely to present special phenotypes. After comparing the phenotypes of patients carrying the five hotspot variants, we revealed that patients carrying c.624dupA (p.C209Mfs*21) not only were associated with later onset of disease and more involved joints but also showed a higher likelihood of elbow involvement. By comparing clinical manifestations among PPRD patients with different genotypes, patients carrying biallelic null variants were more likely to have shoulder joints involved and present long bone shortening with severe deformities. All three patients with long bone shortening and severe deformities carried biallelic null variants, with at least one variant located in exon 2. These null variants may lead to premature termination of *CCN6* translation and protein degradation, thereby contributing to shortening and severe long deformities.

Notably, we observed variable expressivity in PPRD patients. Patients with the same genotype from the same pedigree may show variable phenotypes. As the disease name suggests, the severity of this disease increases as patients age. Younger patients may have fewer involved joints and milder presentations, as exemplified in siblings with long bone deformities. The 3-year-old younger brother presented only deformities in the lower extremities, while the sister showed classic PPRD phenotypes involving multiple joints. The variability in phenotypes may be explained by the presence of modifier genes, as well as environmental triggers.

Sanger sequencing should be prioritized for PPRD screening. During 2007 and 2013, our laboratory at PUMCH performed Sanger sequencing for 42 patients suspected of PPRD and returned positive results for 34 cases and negative results for six cases. Two genetic testing results could not be retrieved. The diagnostic rate was 85% (34/40). We propose that by raising awareness of this disease among clinicians, PPRD can be diagnosed with a high diagnostic rate with Sanger sequencing.

One limitation of this study is the briefness of medical records and the accidental loss of genetic results and medical records. As most PPRD patients at PUMCH were seen in outpatient scenarios, their medical records were often briefly documented. The clinical data from the literature also vary widely in detail, and data from some sources may be too brief to analyze, leading to inevitable bias. Due to the progressive nature of this disease, it would be more advisable to perform stratified analysis by disease-burden years. However, the rarity of PPRD and the briefness of outpatient records limited our analysis within the general patient group.

In eight patients, only a single monoallelic *CCN6* variant was found. As the PCR primers we designed were specifically designed for the five exons and intron‒exon junctions, our Sanger sequencing may fail to capture intronic variants and those on the 5′-UTR. Note that PPRD is typically characterized by autosomal recessive inheritance, and the patients showed classic PPRD phenotypes with a pathogenic variant identified on one allele. We speculate the presence of another pathogenic variant on the other allele that is located in the 5′-UTR or intronic regions.

In summary, through a retrospective analysis of genotypes and phenotypes in 105 Chinese PPRD patients, we characterized a unique genotype spectrum and identified nine novel variants and five hotspot variations among Chinese PPRD patients. We proposed genotype–phenotype associations in variants c.725_726del (p.K242Rfs*36), c.624dupA (p.C209Mfs*21), c.667T>G (p.C223G) and null variants on exon 2, for modifying onset age, joint involvement, and atypical deformities. For suspected PPRD patients with typical clinical features, Sanger sequencing, rather than NGS, should be prioritized.

## Supplementary Information

Below is the link to the electronic supplementary material.

Supplementary file 1 (DOCX 15 kb)

## Data Availability

Data are available from the corresponding author upon reasonable request.
