# Supplementary material for: Unique mutation spectrum of progressive pseudorheumatoid dysplasia in the Chinese population: a retrospective genotype–phenotype analysis of 105 patients
Source: World J Pediatr. 2023 Jan 9;19(7):674–86. doi: 10.1007/s12519-022-00674-7 (PMC10258178; doi:10.1007/s12519-022-00674-7)
Supplement: Supplementary file 1 — (DOCX 15 kb) [file 12519_2022_674_MOESM1_ESM.docx]

**Supplementary Table 1**. PCR primers

| **Primer** | **Sequence (5’-3’)** | **Length (bp)** | **Tm (℃)** |
| --- | --- | --- | --- |
| WISP3E1F | GGAACAGGTAACACAGTCAC | 490 | 60 |
| WISP3E1R | GCCATTGATCCTCCCTCTTC |  |  |
| WISP3E2F | ACCACTCTGTATACTACCTG | 478 | 56 |
| WISP3E2R | CACTCCAAGCTAACAATTGC |  |  |
| WISP3E3F | CATACAGGAGATGATCCGTT | 450 | 56 |
| WISP3E3R | TCTGACCACCAATCAACAAG |  |  |
| WISP3E4F | GAGACTGGACTGTAATACTT | 610 | 56 |
| WISP3E4R | TTAGAATCTGCTCTGGTTGG |  |  |
| WISP3E5F | TGCTGGAAATCACTACATAG | 530 | 54 |
| WISP3E5R | CTGAGAATAGGCATTCATAC |  |  |
